# 2,4,6-Trimethyl­pyridinium nitrate

**DOI:** 10.1107/S1600536810032629

**Published:** 2010-08-21

**Authors:** Shahzad Sharif, Mehmet Akkurt, Islam Ullah Khan, Abdul Rauf, Irfana Mariam

**Affiliations:** a Materials Chemistry Laboratory, Department of Chemistry, Government College University, Lahore 54000, Pakistan; b Department of Physics, Faculty of Sciences, Erciyes University, 38039 Kayseri, Turkey

## Abstract

In the title compound, C_8_H_12_N^+^·NO_3_^−^, the cation lies on a mirror plane and the N and one C atom lie on a twofold axis. In the crystal, the anions and cations are linked by N—H⋯O inter­actions along the *b* axis and a short N—O⋯π contact [3.2899 (5) Å] also occurs.

## Related literature

For the use of *sym*-collidine and its derivatives, see: Brunel & Rousseau (1995 ▶); Homsi & Rousseau (1998 ▶); Rousseau & Robin (1997 ▶); Simonot & Rousseau (1994 ▶); Syper *et al.* (1980 ▶); Yamamoto *et al.* (1992 ▶). For structural properties of the related compound, 2,4,6-collidine, see: Bond & Davies (2001 ▶).
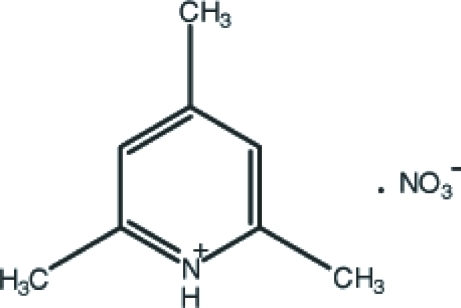


## Experimental

### 

#### Crystal data

C_8_H_12_N^+^·NO_3_^−^
*M* *_r_* = 184.20
Orthorhombic, 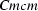

*a* = 9.328 (1) Å
*b* = 15.1327 (13) Å
*c* = 6.4967 (7) Å
*V* = 917.06 (16) Å^3^
*Z* = 4
Mo *K*α radiation
μ = 0.10 mm^−1^
*T* = 296 K
0.28 × 0.16 × 0.07 mm

#### Data collection

Bruker APEXII CCD diffractometer
1839 measured reflections
648 independent reflections
410 reflections with *I* > 2σ(*I*)
*R*_int_ = 0.030

#### Refinement

*R*[*F* ^2^ > 2σ(*F* ^2^)] = 0.047
*wR*(*F* ^2^) = 0.149
*S* = 1.00
648 reflections
58 parameters
8 restraints
All H-atom parameters refined
Δρ_max_ = 0.16 e Å^−3^
Δρ_min_ = −0.19 e Å^−3^

### 

Data collection: *APEX2* (Bruker, 2007 ▶); cell refinement: *SAINT* (Bruker, 2007 ▶); data reduction: *SAINT*; program(s) used to solve structure: *SIR97* (Altomare *et al.*, 1999 ▶); program(s) used to refine structure: *SHELXL97* (Sheldrick, 2008 ▶); molecular graphics: *ORTEP-3 for Windows* (Farrugia, 1997 ▶); software used to prepare material for publication: *WinGX* (Farrugia, 1999 ▶) and *PLATON* (Spek, 2009 ▶).

## Supplementary Material

Crystal structure: contains datablocks global, I. DOI: 10.1107/S1600536810032629/bx2300sup1.cif

Structure factors: contains datablocks I. DOI: 10.1107/S1600536810032629/bx2300Isup2.hkl

Additional supplementary materials:  crystallographic information; 3D view; checkCIF report

## Figures and Tables

**Table 1:** Hydrogen-bond geometry (Å, °)

*D*—H⋯*A*	*D*—H	H⋯*A*	*D*⋯*A*	*D*—H⋯*A*
N1—H1⋯O2^i^	0.875 (18)	2.331 (16)	3.139 (3)	153.7 (2)
N1—H1⋯O2^ii^	0.875 (18)	2.331 (16)	3.139 (3)	153.7 (2)

Symmetry codes: (i) 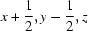; (ii) 
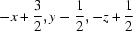
.

**Fig. 1.**
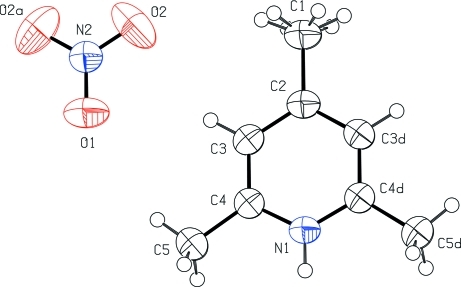
A view of the title molecule. Displacement ellipsoids are drawn at the 50% probability level. [Symmetry codes: (a) 1-x, y, 1/2-z; (d) 2-x, y, 1/2-z.]

**Fig. 2.**
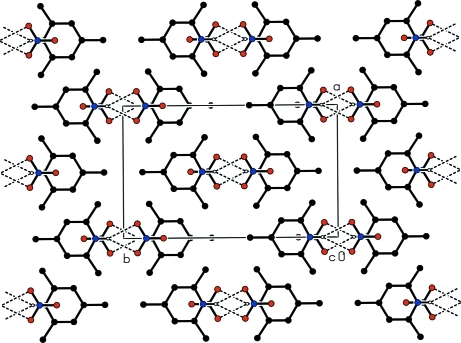
A packing diagram of the title molecule showing the N—H···O interactions, down the c axis. All hydrogen atoms not involved in hydrogen bonding have been omitted for clarity.

## supplementary crystallographic information

### Comment

Sym-collidine and its derivatives are extensively used in organic synthesis (Syper *et al.*, 1980; Rousseau *et al.*, 1997). Bis(2,4,6-trimethylpyridine)iodine(I) and -bromine(I) hexafluorophosphate have been used for specific electrophilic halogenations (Homsi *et al.*, 1998; Simonot *et al.*, 1994; Brunel *et al.*, 1995). It is also used in the synthesis of vitamin D (Yamamoto *et al.*, 1992). Here in we reported the crystal structure of collidinium nitrate.

In the title compound (I), (Fig. 1),the cation  lies on a mirror plane and the N and one C atoms lies on two-fold axis. The anions and cations are linked by N—H···O interactions along the *b* axis. The bond distances and angles in (I) agree with those reported in a similar compound 2,4,6-collidine (Bond & Davies, 2001).

The anions and cations of (I) are linked by N—H···O interactions along the *b* axis (Table 1, Fig. 2). In the crystal structure, the O1 atom in the nitrate anion generates the N—O···π interactions [N2—O1···*Cg*1^iii^ = 3.2899 (5) Å and N2—O1···*Cg*1^iv^ = 3.2899 (5) Å; symmetry codes: (iii) -1/2 + *x*, 1/2 - *y*, -*z*; (iv) -1/2 + *x*, 1/2 - *y*, 1-*z. Cg*1 is a centroid of the aromatic pyridine ring] between two pyridine rings as a sandwich to establish the packing.

### Experimental

To 2 ml of trimethyl pyridine, concentrated nitric acid (2 ml) was added drop wise. The mixture was refluxed for an hour, filtered. Within half an hour needle like crystals of titled compound appeared, suitable for *x*-ray crystallography.

### Refinement

All H atoms were found on the difference map and refined with the distance restraints of N—H = 0.875 (18) Å and C—H = 0.93 (2) - 0.96 (4) Å. Their displacement parameters were constrained to ride on their parent atoms [U_iso_(H) = 1.5U_eq_(C) for methyl H atoms and 1.2U_eq_(C,N) for other atoms].

### Crystal data

**Table:**

C_8_H_12_N^+^·NO_3_^−^	*F*(000) = 392
*M**_r_* = 184.20	*D*_x_ = 1.334 Mg m^−^^3^
Orthorhombic, *C**m**c**m*	Mo *K*α radiation, λ = 0.71073 Å
Hall symbol: -C 2c 2	Cell parameters from 494 reflections
*a* = 9.328 (1) Å	θ = 4.1–23.2°
*b* = 15.1327 (13) Å	µ = 0.10 mm^−^^1^
*c* = 6.4967 (7) Å	*T* = 296 K
*V* = 917.06 (16) Å^3^	Needle, colourless
*Z* = 4	0.28 × 0.16 × 0.07 mm

### Data collection

**Table:**

Bruker APEXII CCD diffractometer	410 reflections with *I* > 2σ(*I*)
Radiation source: sealed tube	*R*_int_ = 0.030
graphite	θ_max_ = 28.3°, θ_min_ = 4.1°
φ and ω scans	*h* = −11→12
1839 measured reflections	*k* = −19→20
648 independent reflections	*l* = −8→4

### Refinement

**Table:**

Refinement on *F*^2^	Primary atom site location: structure-invariant direct methods
Least-squares matrix: full	Secondary atom site location: difference Fourier map
*R*[*F*^2^ > 2σ(*F*^2^)] = 0.047	Hydrogen site location: inferred from neighbouring sites
*wR*(*F*^2^) = 0.149	All H-atom parameters refined
*S* = 1.00	*w* = 1/[σ^2^(*F*_o_^2^) + (0.0764*P*)^2^ + 0.2375*P*] where *P* = (*F*_o_^2^ + 2*F*_c_^2^)/3
648 reflections	(Δ/σ)_max_ < 0.001
58 parameters	Δρ_max_ = 0.16 e Å^−^^3^
8 restraints	Δρ_min_ = −0.19 e Å^−^^3^

### Special details

**Table:**

Geometry. Bond distances, angles *etc*. have been calculated using the rounded fractional coordinates. All su's are estimated from the variances of the (full) variance-covariance matrix. The cell e.s.d.'s are taken into account in the estimation of distances, angles and torsion angles
Refinement. Refinement on *F*^2^ for ALL reflections except those flagged by the user for potential systematic errors. Weighted *R*-factors *wR* and all goodnesses of fit *S* are based on *F*^2^, conventional *R*-factors *R* are based on *F*, with *F* set to zero for negative *F*^2^. The observed criterion of *F*^2^ > σ(*F*^2^) is used only for calculating -*R*-factor-obs *etc*. and is not relevant to the choice of reflections for refinement. *R*-factors based on *F*^2^ are statistically about twice as large as those based on *F*, and *R*-factors based on ALL data will be even larger.

### Fractional atomic coordinates and isotropic or equivalent isotropic displacement parameters (Å^2^)

**Table:**

	*x*	*y*	*z*	*U*_iso_*/*U*_eq_	Occ. (<1)
N1	1.00000	0.13163 (15)	0.25000	0.0411 (8)	
C1	1.00000	0.4105 (2)	0.25000	0.0604 (13)	
C2	1.00000	0.31135 (19)	0.25000	0.0444 (10)	
C3	0.8725 (2)	0.26452 (14)	0.25000	0.0454 (7)	
C4	0.8728 (2)	0.17377 (14)	0.25000	0.0422 (7)	
C5	0.7402 (3)	0.11973 (17)	0.25000	0.0577 (9)	
O1	0.50000	0.31452 (15)	0.25000	0.0754 (10)	
O2	0.6109 (2)	0.43578 (17)	0.25000	0.1128 (13)	
N2	0.50000	0.39433 (16)	0.25000	0.0469 (9)	
H1	1.00000	0.0738 (12)	0.25000	0.0560*	
H1A	1.095 (3)	0.435 (4)	0.25000	0.0700*	0.500
H1B	0.949 (3)	0.432 (2)	0.369 (4)	0.0700*	0.500
H3	0.7858 (19)	0.2953 (14)	0.25000	0.0560*	
H5A	0.661 (2)	0.1569 (14)	0.25000	0.0700*	
H5B	0.7366 (19)	0.0801 (10)	0.135 (3)	0.0700*	

### Atomic displacement parameters (Å^2^)

**Table:**

	*U*^11^	*U*^22^	*U*^33^	*U*^12^	*U*^13^	*U*^23^
N1	0.0464 (14)	0.0334 (12)	0.0436 (16)	0.0000	0.0000	0.0000
C1	0.065 (2)	0.0383 (16)	0.078 (3)	0.0000	0.0000	0.0000
C2	0.0526 (17)	0.0367 (14)	0.044 (2)	0.0000	0.0000	0.0000
C3	0.0451 (11)	0.0428 (12)	0.0483 (15)	0.0043 (9)	0.0000	0.0000
C4	0.0428 (11)	0.0432 (11)	0.0407 (14)	−0.0009 (9)	0.0000	0.0000
C5	0.0470 (13)	0.0491 (13)	0.077 (2)	−0.0054 (10)	0.0000	0.0000
O1	0.098 (2)	0.0401 (12)	0.088 (2)	0.0000	0.0000	0.0000
O2	0.0894 (16)	0.1011 (18)	0.148 (3)	−0.0507 (13)	0.0000	0.0000
N2	0.0526 (15)	0.0461 (15)	0.0420 (17)	0.0000	0.0000	0.0000

### Geometric parameters (Å, °)

**Table:**

O1—N2	1.208 (3)	C1—H1A^i^	0.96 (4)
O2—N2	1.210 (2)	C1—H1A	0.96 (4)
N1—C4	1.347 (2)	C1—H1B	0.96 (3)
N1—C4^i^	1.347 (2)	C1—H1B^ii^	0.96 (3)
N1—H1	0.875 (18)	C1—H1B^i^	0.96 (3)
C1—C2	1.500 (4)	C1—H1B^iii^	0.96 (3)
C2—C3^i^	1.384 (2)	C3—H3	0.933 (19)
C2—C3	1.384 (2)	C5—H5B^iii^	0.959 (18)
C3—C4	1.373 (3)	C5—H5A	0.93 (2)
C4—C5	1.483 (3)	C5—H5B	0.959 (18)
			
O2···N1^iv^	3.139 (3)	H1···H5B^i^	2.570 (18)
O2···C5^v^	3.111 (4)	H1···H5B^iii^	2.570 (18)
O2···N1^v^	3.139 (3)	H1···H5B	2.570 (18)
O1···H3	2.682 (18)	H1···O2^ix^	2.331 (16)
O1···H5A^vi^	2.82 (2)	H1···N2^ix^	2.716 (18)
O1···H3	2.682 (18)	H1···H5B^ii^	2.570 (18)
O1···H5A	2.82 (2)	H1···O2^xii^	2.331 (16)
O1···H3^vi^	2.682 (18)	H1···O2^xvi^	2.331 (16)
O1···H3^vii^	2.682 (18)	H1···N2^xvi^	2.716 (18)
O1···H5A^vii^	2.82 (2)	H1···N2^xiii^	2.716 (18)
O1···H5A	2.82 (2)	H1···N2^xii^	2.716 (18)
O2···H1^iv^	2.331 (16)	H1···O2^xiii^	2.331 (16)
O2···H5B^viii^	2.888 (19)	H1A···H3^i^	2.39 (6)
O2···H3	2.68 (2)	H1A···O2^i^	2.74 (3)
O2···H1A^i^	2.74 (3)	H1A···O2^ii^	2.74 (3)
O2···H3	2.68 (2)	H1A···H3^i^	2.39 (6)
O2···H1A^i^	2.74 (3)	H1B···H5B^viii^	2.45 (3)
O2···H1^v^	2.331 (16)	H3···O1	2.682 (18)
O2···H5B^v^	2.711 (16)	H3···O2	2.68 (2)
N1···N2^viii^	3.2720 (5)	H3···H5A	2.40 (3)
N1···O2^ix^	3.139 (3)	H3···O1	2.682 (18)
N1···N2^x^	3.2720 (5)	H3···O2	2.68 (2)
N1···N2^xi^	3.2720 (5)	H3···H1A^i^	2.39 (6)
N1···O2^xii^	3.139 (3)	H3···O1	2.682 (18)
N1···O2^xiii^	3.139 (3)	H3···O1	2.682 (18)
N1···N2^xiv^	3.2720 (5)	H3···H1A^i^	2.39 (6)
N1···N2^xv^	3.2720 (5)	H5A···O1	2.82 (2)
N1···O2^xvi^	3.139 (3)	H5A···O1	2.82 (2)
N1···N2^xvii^	3.2720 (5)	H5A···H3	2.40 (3)
N1···N2^xviii^	3.2720 (5)	H5A···O1	2.82 (2)
N1···N2^xix^	3.2720 (5)	H5A···O1	2.82 (2)
N2···N1^viii^	3.2720 (5)	H5B···O2^x^	2.888 (19)
N2···N1^x^	3.2720 (5)	H5B···H1B^x^	2.45 (3)
N2···H1^iv^	2.716 (18)	H5B···H1	2.570 (18)
N2···H1^v^	2.716 (18)	H5B···O2^xix^	2.888 (19)
C5···O2^xiii^	3.111 (4)	H5B···O2^xiii^	2.711 (16)
C5···O2^xvi^	3.111 (4)	H5B···O2^xvi^	2.711 (16)
			
C4—N1—C4^i^	123.5 (2)	H1A^i^—C1—H1B	54.2 (17)
C4—N1—H1	118.26 (12)	H1B—C1—H1B^i^	141 (3)
C4^i^—N1—H1	118.26 (12)	H1B—C1—H1B^iii^	107 (2)
O2—N2—O2^vi^	117.5 (3)	H1A—C1—H1A^i^	135 (5)
O1—N2—O2^vi^	121.23 (15)	H1A—C1—H1B^i^	54.2 (17)
O1—N2—O2	121.23 (15)	H1A^i^—C1—H1B^iii^	54.2 (17)
C1—C2—C3	120.79 (13)	H1A^i^—C1—H1B^ii^	109 (2)
C1—C2—C3^i^	120.79 (13)	H1B^i^—C1—H1B^iii^	59 (2)
C3—C2—C3^i^	118.4 (2)	H1B^i^—C1—H1B^ii^	107 (2)
C2—C3—C4	120.67 (19)	H1B^iii^—C1—H1B^ii^	141 (3)
N1—C4—C5	118.3 (2)	C2—C1—H1A	113 (3)
C3—C4—C5	123.35 (19)	H1B—C1—H1B^ii^	59 (2)
N1—C4—C3	118.37 (18)	H1A^i^—C1—H1B^i^	109 (2)
C2—C1—H1A^i^	113 (3)	C2—C3—H3	119.3 (13)
C2—C1—H1B^i^	109.7 (18)	C4—C3—H3	120.1 (13)
C2—C1—H1B^ii^	109.7 (18)	C4—C5—H5B^iii^	112.0 (11)
H1A—C1—H1B	109 (2)	H5A—C5—H5B^iii^	110.6 (13)
C2—C1—H1B^iii^	109.7 (18)	H5B—C5—H5B^iii^	102.4 (15)
C2—C1—H1B	109.7 (18)	H5A—C5—H5B	110.6 (13)
H1A—C1—H1B^iii^	109 (2)	C4—C5—H5A	109.2 (13)
H1A—C1—H1B^ii^	54.2 (17)	C4—C5—H5B	112.0 (11)
			
C1—C2—C3—C4	180.00	C2—C3—C4—C5	180.00
C2—C3—C4—N1	0.00		

Symmetry codes: (i) −*x*+2, *y*, −*z*+1/2; (ii) −*x*+2, *y*, *z*; (iii) *x*, *y*, −*z*+1/2; (iv) *x*−1/2, *y*+1/2, *z*; (v) −*x*+3/2, *y*+1/2, −*z*+1/2; (vi) −*x*+1, *y*, −*z*+1/2; (vii) −*x*+1, *y*, *z*; (viii) −*x*+3/2, −*y*+1/2, *z*+1/2; (ix) *x*+1/2, *y*−1/2, *z*; (x) −*x*+3/2, −*y*+1/2, *z*−1/2; (xi) −*x*+3/2, −*y*+1/2, −*z*+1; (xii) *x*+1/2, *y*−1/2, −*z*+1/2; (xiii) −*x*+3/2, *y*−1/2, *z*; (xiv) *x*+1/2, −*y*+1/2, *z*−1/2; (xv) *x*+1/2, −*y*+1/2, *z*+1/2; (xvi) −*x*+3/2, *y*−1/2, −*z*+1/2; (xvii) *x*+1/2, −*y*+1/2, −*z*; (xviii) *x*+1/2, −*y*+1/2, −*z*+1; (xix) −*x*+3/2, −*y*+1/2, −*z*.

### Hydrogen-bond geometry (Å, °)

**Table:**

*D*—H···*A*	*D*—H	H···*A*	*D*···*A*	*D*—H···*A*
N1—H1···O2^ix^	0.875 (18)	2.331 (16)	3.139 (3)	153.7 (2)
N1—H1···O2^xvi^	0.875 (18)	2.331 (16)	3.139 (3)	153.7 (2)

Symmetry codes: (ix) *x*+1/2, *y*−1/2, *z*; (xvi) −*x*+3/2, *y*−1/2, −*z*+1/2.
